# Dpep2 Emerging as a Modulator of Macrophage Inflammation Confers Protection Against CVB3-Induced Viral Myocarditis

**DOI:** 10.3389/fcimb.2019.00057

**Published:** 2019-03-07

**Authors:** Xiaoli Yang, Yan Yue, Sidong Xiong

**Affiliations:** Jiangsu Key Laboratory of Infection and Immunity, Institutes of Biology and Medical Sciences, Soochow University, Suzhou, China

**Keywords:** coxsackievirus B3 (CVB3), viral myocarditis (VMC), Dpep2, macrophages, NF-κB signaling

## Abstract

Overwhelming cardiac inflammation has been reported to be the pathogenic mechanism of Coxsackievirus B3 (CVB3)-induced viral myocarditis (VMC), while the detailed molecular mechanisms remain unknown. Membrane-bound dipeptidases (MBD, also known as Dpep) have been shown to be involved in inflammatory diseases. However, the clear and direct evidence of their impacts on inflammation is still lacking. In this study, our results revealed that Dpep2 expression was remarkably increased during CVB3 infection, and primarily produced by the cardiac tissue-infiltrating macrophages instead of constitutive cardiomyocytes. Macrophages have been reported to play an important pathological role in driving VMC. Interestingly, macrophage-specific Dpep2 deletion robustly aggravated CVB3-induced cardiac inflammation, evidenced by augmented expression of TNF-α, IL-6, and MCP-1 in heart tissue. In addition, Dpep2-deficient bone-marrow derived macrophages (BMDMs) generated more TNF-α, IL-6, and MCP-1 after CVB3 stimulation compared with the control BMDMs. Moreover, this suppressive effect of Dpep2 on macrophages relied on its repression on NF-κB signaling pathway, but not on its conventional hydrolysate LTE4. Taken together, this study revealed that Dpep2 could protect against CVB3-induced VMC by acting as a suppressor of macrophage inflammation. Better understanding how macrophage Dpep2 dampened the cardiac inflammation would provide us with insights for the efficient control of CVB3-induced VMC.

## Introduction

Viral myocarditis (VMC) is a virus-induced cardiac inflammatory disease, which often manifests as acute myocarditis, and could also further develop to chronic VMC, dilated cardiomyopathy, and even heart failure. Coxsackievirus B3 (CVB3), a non-enveloped, positive-sense, single-stranded RNA virus, belongs to the genus Enterovirus, which is considered as an important causative agent for VMC (Feldman and Mcnamara, 2000; Esfandiarei and Mcmanus, 2008; Garmaroudi et al., 2015). However, to date, no effective and specific treatments against CVB3-induced VMC are available because of the poor understanding of its pathogenic mechanisms. A wide range of clinical and experimental studies indicate that besides of virus-mediated damage, immune pathological cardiac injury is an important pathogenic mechanism of CVB3-induced VMC (Westermann et al., 2010). Therefore, immunomodulation has become one of the most promising prophylactic and therapeutic strategies against this disease (Marchant and Mcmanus, 2010; Papageorgiou et al., 2012; Valaperti et al., 2013). Elucidating the mechanisms of over-reactive cardiac immune responses would shed light on better understanding the key pathogenic processes of CVB3-induced VMC, and provide some clues to the novel therapeutic strategy development.

Membrane-bound dipeptidase (Dpep) family, including 3 members (Dpep1, Dpep2, and Dpep3), is anchored by glycosylphosphatidylinositol (GPI), and responsible for hydrolysis of dipeptides including leukotriene D4, the beta-lactam ring of some antibiotics, and cystinyl-bis-glycine (cys-bis-gly) formed during glutathione degradation. Recently, Dpep expression has been reported to be changed in the context of inflammatory diseases (Chen et al., 2015; Dabritz et al., 2015), suggesting their potential association with immune responses.

Here, we found that cardiac Dpep2 expression was significantly increased in CVB3-induced myocarditis, suggesting its possible involvement in VMC. Herein we focused on exploring the potential roles of Dpep2 in CVB3-induced VMC.

## Materials and Methods

### Mice and CVB3 Infection

Male BALB/c (6–8 weeks) mice were obtained from the Experimental Animal Center of the Chinese Academy of Sciences (Shanghai, China) and kept in the Animal Center of Soochow University. The Dpep2^fl/fl^ mice were purchased from the Model Animal Research Center of Nanjing University, and were crossed to LyzM-Cre^+^ mice to generate the macrophage-specific Dpep2-deficient (Dpep2^fl/fl^ LyzM-Cre^+^) mice. All animal experiments were performed in accordance with the guidelines for the Care and Use of Laboratory Animals (Ministry of Health, China, 1998). All animal experiment protocols in this study were approved by the Ethics Committee of Soochow University. Mice were infected with CVB3 via intraperitoneal (i.p.) injections at the dose of 10^3^ PFUs. CVB3 (Nancy strain) was passaged in HeLa cells (ATCC catalog number:CCL-2).

### Cell Isolation, Preparation, and Culture

Bone-marrow derived macrophages (BMDMs) were prepared as previously described (Bauerfeld et al., 2012). Briefly, freshly isolated bone marrow cells were plated in RPMI 1640, containing 30% L929-conditioned medium, 10% FBS, 2 mM L-glutamine, 100 U/mL penicillin, and 100 μg/mL streptomycin for 6 days. The purity of induced BMDMs was measured by FACS using anti-F4/80 antibody (BD Biosciences) and anti-CD11b monoclonal antibody (BD Biosciences). The purity was >90%. Cardiac tissue-infiltrating macrophages were prepared by FACS sorting using anti-F4/80 and anti-CD11b antibodies as previously described (Liu et al., 2014).

### RNA Isolation, RT-PCR, and Real-Time PCR

Total RNA was isolated using an RNeasy Mini RNA Isolation Kit (QIAGEN) and subjected to RT-PCR with cDNA Synthesis Kit (Takara). Real-time PCR was performed with the SYBR Green PCR Master Mix kit (Takara) following the manufacturer's protocol. The transcripts were quantified by real-time PCR and normalized to the amount of GAPDH mRNA. The primers were listed as follow: CVB3 Forward: 5′-ATCAAGTTGCGTGCTGTG-3′; CVB3 Rev: 5′-TGCGAAATGAAAGGA GTGT-3′; IL-6 Forward: 5′-ACAACCACGGCCTTCCCTACTT-3′; IL-6 Rev: 5′-CACGATTTCCCAG AGAACATGTG-3′; MCP-1 Forward: 5′-CCCACTCAC CTGCTGCTACT-3′; MCP-1 Rev: 5′-TCTGGACCCATTCCTTCTTG-3′; GAPDH Forward: 5′-GAGCCAAACGGGTCATCATCT-3′; GAPDH Rev: 5′- GAGGGGCCAT CCACAGTCTT-3′. The reverse transcription primer for CVB3 negative strand: 5′-GCGAAGAGTCTATTGAGCTA-3′, CVB3 positive strand: 5′-CACCGGATGGCCAATCCA-3′. The target gene expression was normalized to GAPDH expression by the 2^−ΔΔ*Ct*^ method.

### ELISAs

Heart tissue homogenates were prepared by liquid nitrogen milling and suspended at a concentration of 100 mg/mL in lysis buffer for 30 min at 4°C and then centrifuged for 10 min. Expression of TNF-α, IL-6, and MCP-1 in cell culture supernatants and heart tissue homogenates were determined using enzyme-linked immunosorbent assay (ELISA) kit (eBioscience). The LTE4 level was determined by the LTE4 ELISA Kit (Cayman). The cTnI level in serum was detected by ELISA (Life Diagnostics, USA).

### Echocardiography

Cardiac functions were assessed with an echocardiography system (Vevo2100, Visual Sonics, Canada) on day 7 post CVB3 infection based on left ventricular ejection fraction (LVEF) and left ventricular fractional shortening (LVFS) as previously described (Chai et al., 2014).

### Histopathological Analysis and Myocarditis Scoring

Hearts were harvested, fixed in 10% phosphate-buffered formalin, embedded in paraffin and cut into 5-μm thick sections. Hematoxylin and eosin (H&E) staining was used to identify the level of myocardial inflammation. Sections were examined by two independent investigators in a blinded manner, and the severity of myocarditis was assessed as the percentage of inflamed area over the total area of the heart tissue section with the aid of a microscope eyepiece grid as previously described (Zhang et al., 2017).

### Western Blot

Heart homogenates or cells lysates were subjected to 10% sodium dodecyl polyacrylamide gel electrophoresis separation. The membranes were probed with the primary antibodies anti-Dpep2 (abcam), anti-phosphorylated-p65 (p-p65), anti-p65, anti-IκB-α, anti-phosphorylated-IκB-α, anti-Ikk-α, anti-phosphorylated-Ikk-α (p-Ikk-α) (Cell Signaling Technology) and anti-GAPDH (Sigma). HRP-conjugated anti-rabbit IgG antibody (Southern Biotech) was used as the secondary antibody. Detection was performed by enhanced cheminescence (Pierce; Thermo Fisher Scientific) and band intensities were quantified by ImageJ software.

### Statistical Analysis

All data were analyzed by Prism 6 software (GraphPad Prism 6) and were presented as the means±S.E.M. For analyzing statistical significance between two groups, a Student's t test was used. For analyzing statistical significance between multiple groups, a one-way ANOVA was used. For analyzing multiple time points experiment, a two-way ANOVA was used, *p* < 0.05 was considered as statistically significant.

## Results

### Cardiac Dpep2 Was Significantly Increased in the CVB3-Induced Viral Myocarditis

**Figure 1:**
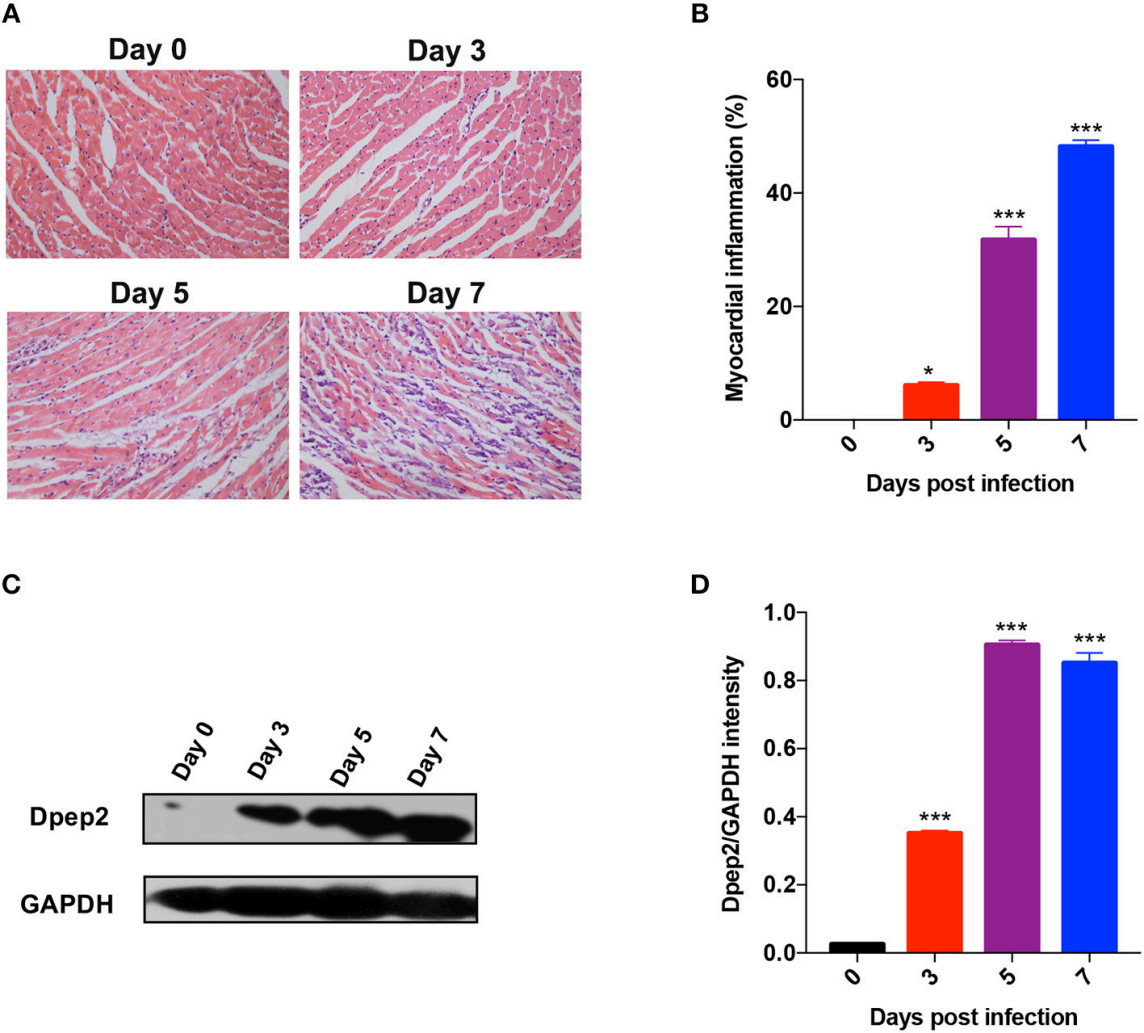
Dpep2 was significantly increased in viral myocarditis (VMC). Male BALB/c mice were intraperitoneally injected with 10^3^ PFU CVB3 and the heart tissues were collected at the indicated time points. Myocarditis severity was evaluated by **(A)** pathological observation (magnification: 200 ×) and **(B)** percentage of myocardial inflammation. **(C)** Myocardial Dpep2 kinetics during CVB3 infection were detected by Western blot. **(D)** Band intensities were quantified by ImageJ software and normalized to GAPDH. Individual experiments were conducted three times with similar results and the representative data were shown. Data were presented as the means ± S.E.M. ^*^p < 0.05; ^***^p < 0.001.

By analyzing Gene expression array database (https://www.ebi.ac.uk/arrayexpress), we found that cardiac Dpep2 mRNA level was increased in CVB3-infected mice compared with the control mice (about 2~fold, data not shown), while the levels of cardiac Dpep1 and Dpep3 in the CVB3-infected mice were comparable to those of the uninfected mice. To verify this result, mice were infected with CVB3 and myocarditis severity was monitored by pathological observation and evaluated by myocardial inflammation percentages. As shown in Figures 1A,B, myocarditis began to appear at day 3, and as time went by, myocarditis further aggravated and massive myocardial inflammation and injury could be observed at day 7 post-infection. This time course of pathological changes was highly coincidence with the dynamic of cardiac Dpep2 expression as shown in Figures 1C,D, in which Dpep2 expression was remarkably increased on day 3 and maintained at the high level from day 5 to day 7, further confirming that CVB3 infection could up-regulate the cardiac Dpep2 expression, and also suggesting that Dpep2 might be involved in the pathogenesis of CVB3-induced myocarditis.

### Macrophage Dpep2 Potently Protected Mice Against Severe Viral Myocarditis

**Figure 2:**
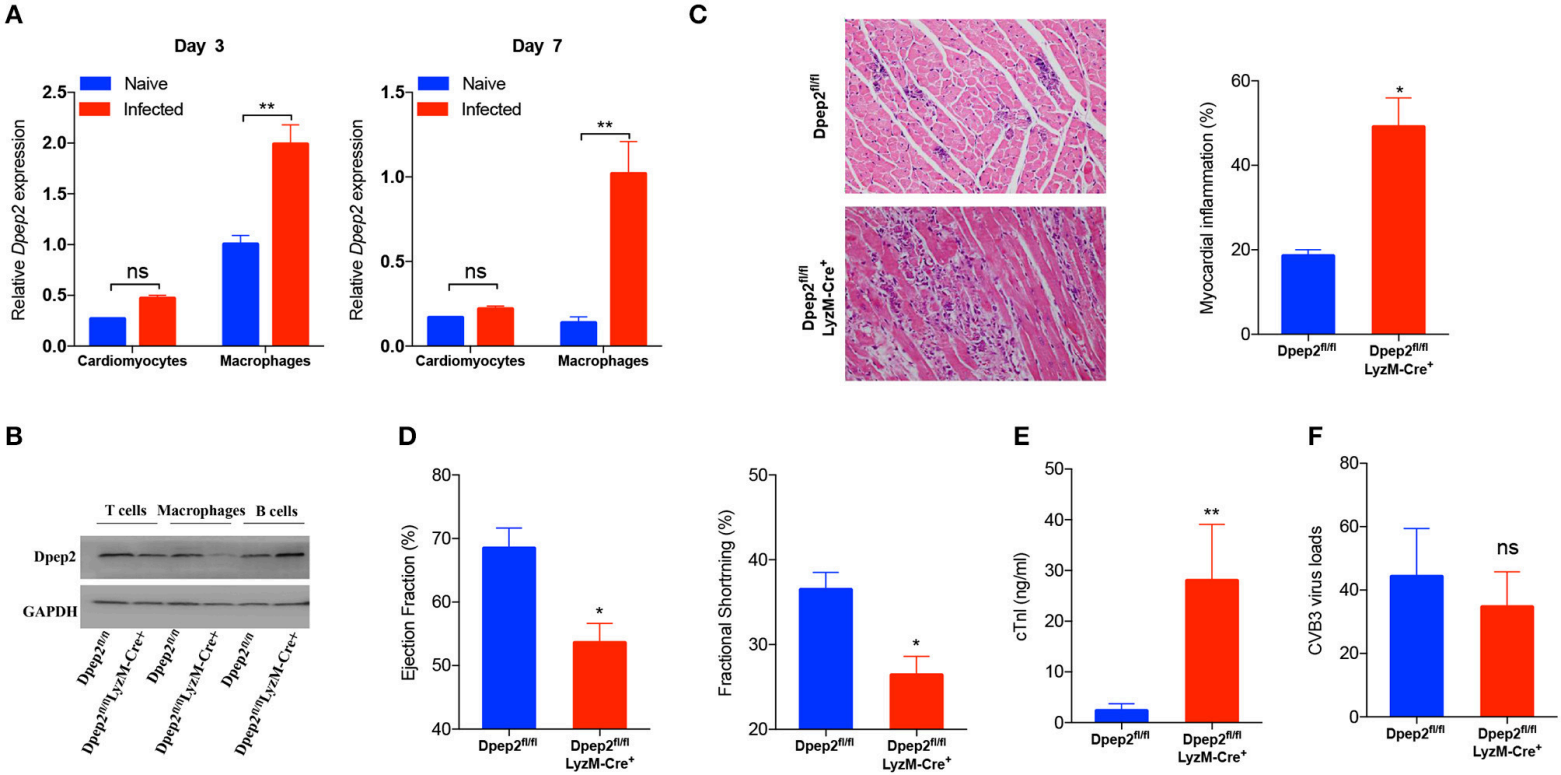
Macrophage Dpep2 deficiency could remarkably aggravate viral myocarditis. **(A)** Dpep2 expression in the cardiomyocytes and tissue-infiltrating macrophages was examined by real-time PCR on day 3 and 7 post CVB3 infection. **(B)** The Dpep2 expression in T cells, B cells, and BMDMs from Dpep2^fl/fl^ and Dpep2^fl/fl^LyzM-Cre^+^ mice were measured by Western blot. **(C)** Myocarditis severity was evaluated by pathological observation (magnification: 200 ×) and percentage of myocardial inflammation. **(D)** Ventricular systolic function was measured using Left ventricular ejection fraction (LVEF) and Left ventricular fractional shortening (LVFS) on day 7. **(E)** The serum cTnI levels were detected by ELISA. **(F)** Viral loads in the myocardium was detected by real-time PCR. Individual experiments were conducted three times with similar results and the representative data were shown. Data were presented as the means ± S.E.M. ^*^p < 0.05; ^**^p < 0.01; ns, no significance.

We next sought to identify the cell source of cardiac Dpep2. Considering that the increase of cardiac Dpep2 appeared on day 3 post-infection, at that time cardiac macrophages began to enrich and represent the primary infiltrating inflammatory cells (Fairweather and Rose, 2007), so we considered macrophages as one of candidates. Although CVB3 could not efficiently infect macrophages (Girn et al., 2002; Zhang et al., 2017), but it could robustly stimulate these cells to produce cytokines and other proteins (Francisco et al., 2018). Therefore, we isolated the cardiomyocytes and the cardiac infiltrating macrophages on day 3 and detected Dpep2 expression. We found that after infection, Dpep2 was significantly increased in macrophages but not in the infected cardiomyocytes, which lasted to day 7 post-infection (Figure 2A). This inspired us that Dpep2 may affect macrophage inflammatory responses.

**Figure 3:**
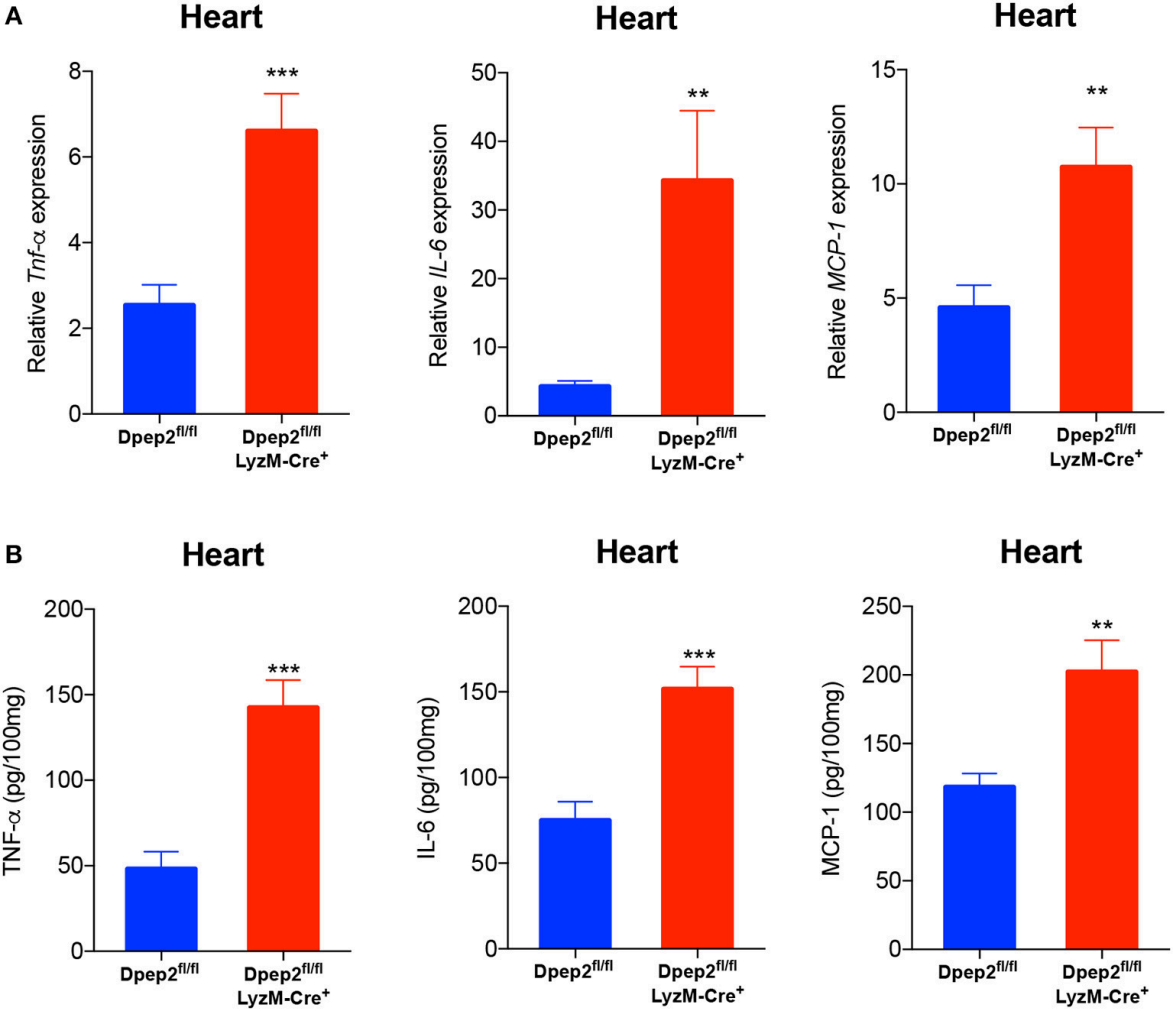
Conditional knockout of Dpep2 in macrophages could markedly promote pro-inflammatory cytokine production *in vivo*. **(A)** Heart tissue homogenates were prepared on day 7 post CVB3 infection, and the levels of pro-inflammatory cytokines including TNF-α, IL-6, and MCP-1 were detected by real-time PCR. **(B)** Heart tissue homogenates were prepared on day 7, and the levels of pro-inflammatory cytokines including TNF-α, IL-6, and MCP-1 were detected by ELISA. Individual experiments were conducted three times with similar results and the representative data were shown. Data were presented as the means ± S.E.M. ^**^p < 0.01; ^***^p < 0.001.

**Figure 4:**
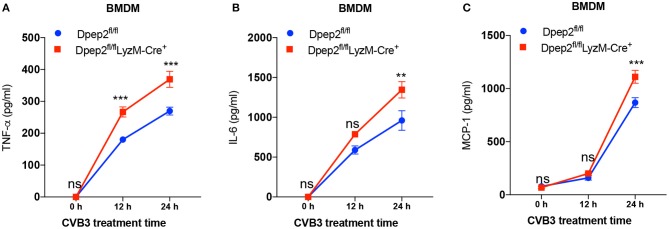
Dpep2 inhibited pro-inflammatory cytokine production by macrophages. BMDMs derived from Dpep2^fl/fl^ LyzM-Cre^+^ or Dpep2^fl/fl^ mice were treated with CVB3 (MOI = 10) for 12, 24 h. **(A–C)** Levels of pro-inflammatory cytokines TNF-α, IL-6, and MCP-1 in the supernatants were detected by ELISA. Individual experiments were conducted three times with similar results and the representative data were shown. Data were presented as the means ± S.E.M. ^**^p < 0.01; ^***^p < 0.001; ns, no significance.

To verify this hypothesis, macrophage-specific Dpep2-deficient mice (Dpep2^fl/fl^ LyzM-Cre^+^) were generated by crossing Dpep2^fl/fl^ mice to LyzM-Cre^+^ mice. Firstly, we confirmed whether Dpep2 was efficiently and specifically deleted in macrophages. For this purpose, T cells, B cells and BMDMs derived from Dpep2^fl/fl^ and Dpep2^fl/fl^ LyzM-Cre^+^ mice were subjected to western blot. As shown in Figure 2B, Dpep2 was specifically deleted in macrophages. Then these mice were infected with CVB3. Excitingly, Dpep2^fl/fl^ LyzM-Cre^+^ mice exhibited more severe myocarditis than the control mice (Dpep2^fl/fl^) as shown by the aggravated cardiac inflammation (Figure 2C), weaker left ventricular systolic function (Figure 2D), higher cTnI activity (Figure 2E) as well as much more abundant pro-inflammatory cytokine production (Figures 3A,B). These results demonstrated that macrophage Dpep2 could protect mice from the extensive cardiac inflammatory responses without obviously impacting cardiac viral loads, as similar cardiac viral loads were evidenced in these two groups (Figure 2F). We also found that *in vitro* stimulation of Dpep2-deficient macrophages with CVB3 could induce more intense production of inflammatory cytokines (TNF-α, IL-6, and MCP-1) (Figures 4A–C) compared with the control macrophages. These results suggest that macrophage Dpep2 protected mice against CVB3-induced VMC by suppressing inflammatory cytokine production.

### Anti-inflammatory Property of Dpep2 Did Not Depend on Its Conventional Hydrolysate LTE4

**Figure 5:**
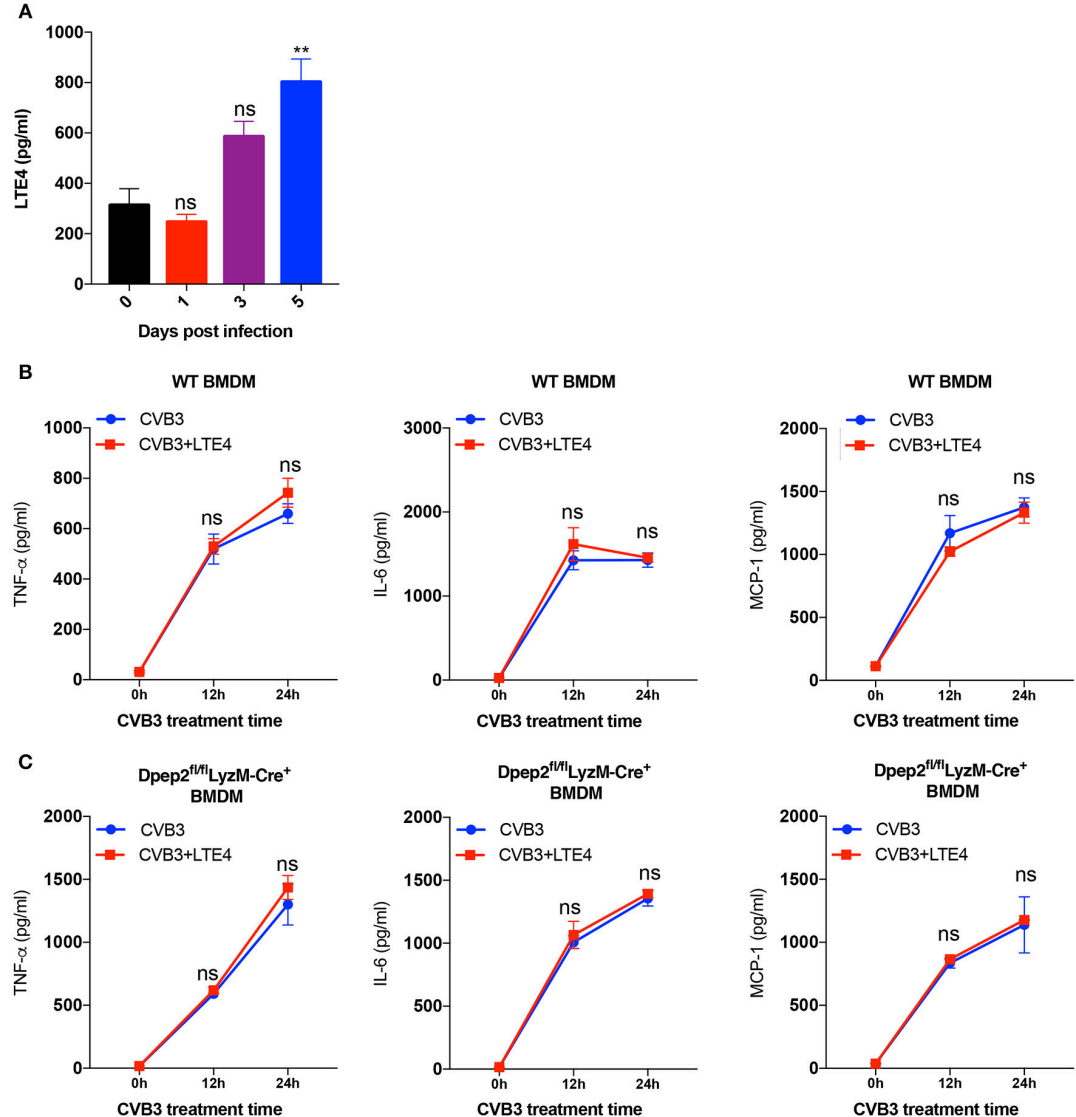
Anti-inflammatory properties of Dpep2 did not rely on the conventional hydrolysate LTE4. **(A)** The kinetics of myocardial LTE4 were detected by ELISA on days 0, 1, 3, 5 post CVB3-infection. **(B)** WT BMDMs were incubated with CVB3 (MOI = 10) and LTE4 (100 nM) for 12 or 24 h. Levels of the pro-inflammatory cytokines TNF-α, IL-6, and MCP-1 in the supernatants were detected by ELISA. **(C)** Dpep2-deficient BMDMs were incubated with CVB3 (MOI = 10) and LTE4 (100 nM) for 12 or 24 h. Levels of the pro-inflammatory cytokines TNF-α, IL-6, and MCP-1 in the supernatants were detected by ELISA. Individual experiments were conducted three times with similar results and the representative data were shown. Data were presented as the means ± S.E.M. ^**^p < 0.01; ns, no significance.

Next, we would like to investigate how Dpep2 regulated the macrophage inflammation. Since leukotriene D4 (LTD4), the substrate of Dpep2, and its hydrolysate LTE4 have been reported to act as pro-inflammatory lipid mediators (Habib et al., 2003; Steinke et al., 2014; Bankova et al., 2016), and we observed the increase of cardiac LTE4 level in mice with CVB3-induced myocarditis (Figure 5A), we firstly deduced that Dpep2 might modulate macrophage inflammation by LTE4. When exogenous LTE4 was added into the WT BMDMs culture system, to our surprise, no obvious augmented pro-inflammatory cytokine production was observed after CVB3 stimulation (Figure 5B), indicating that LTE4 could not limit CVB3-induced macrophage inflammation, and its up-regulation was most likely an accompanying phenomenon. Additionally, we added the exogenous LTE4 to Dpep2-deficient macrophages and detected the production of pro-inflammatory cytokines (TNF-α, IL-6, and MCP-1). As shown in Figure 5C, exogenous LTE4 had no significant impact on CVB3-induced pro-inflammatory cytokines production, further confirming that the anti-inflammatory property of Dpep2 did not depend on its conventional hydrolysate LTE4. Therefore, we concluded that the protective immune suppressive effect of macrophage Dpep2 in VMC did not depend on LTE4.

### Dpep2 Limited CVB3-Induced Macrophage Inflammation by Repressing NF-κB Signaling

**Figure 6:**
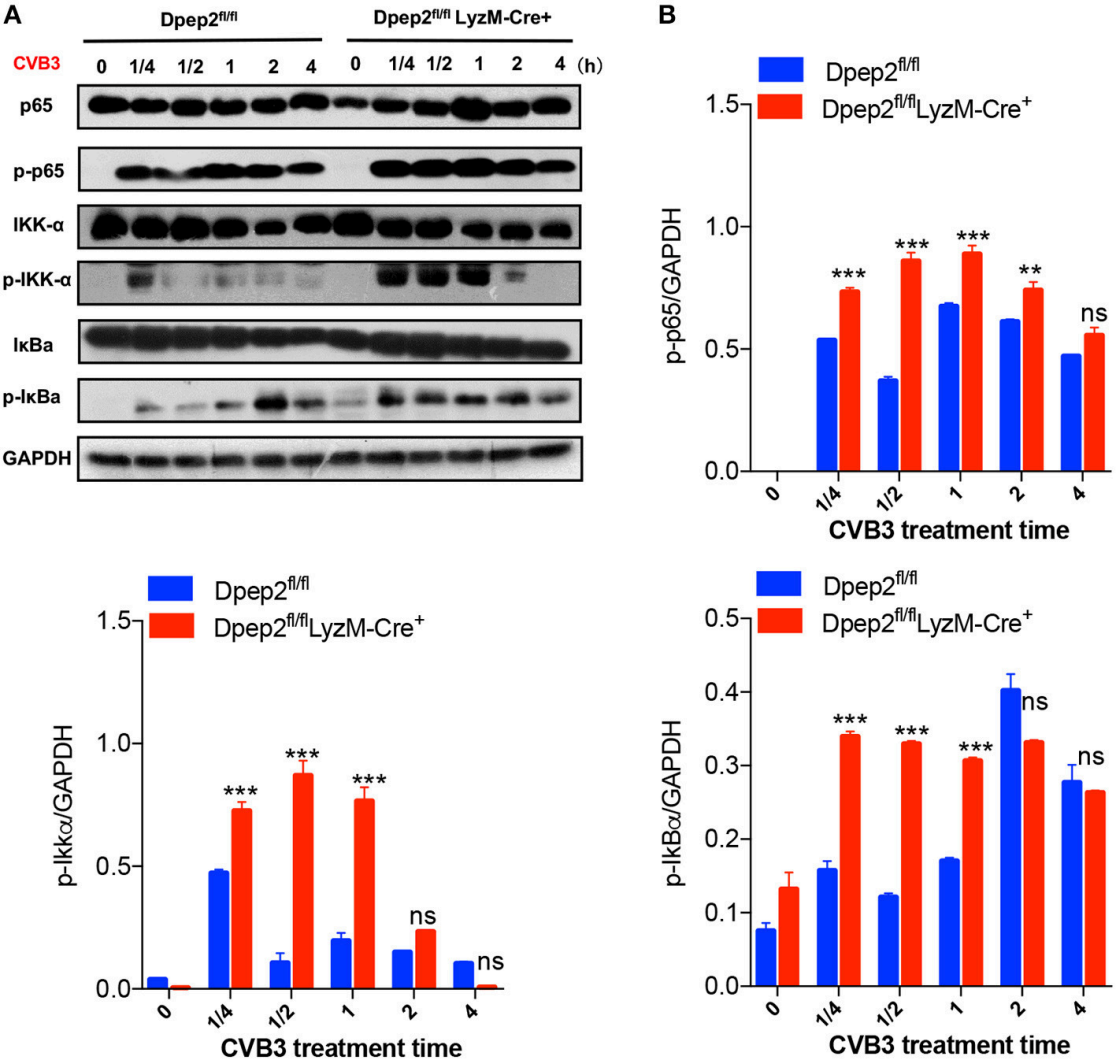
Dpep2 repressed pro-inflammatory responses in macrophages by modulating NF-κB signaling. **(A)** BMDMs derived from Dpep2^fl/fl^ LyzM-Cre^+^ or Dpep2^fl/fl^ mice were treated with CVB3 (MOI = 10) for 15, 30, 60, 120, or 240 min, and the levels of p65, IKKα, IκB, p-p65, p-IKKα, and p-IκB were analyzed by Western blot. **(B)** Band intensities were quantified by ImageJ software and normalized to GAPDH. Data were from one representative experiment among two independent experiments with similar results and were presented as the means ± S.E.M. ^**^p < 0.01; ^***^p < 0.001; ns, no significance.

Considering that the expression of pro-inflammatory cytokines (TNF-α, IL-6, and MCP-1) in macrophage were controlled by the IKK/NF-κB signaling pathway, which plays a pivotal role in initiating, maintaining, and augmenting macrophage inflammatory responses, therefore we tried to detect whether Dpep2 could modulate NF-κB pathway. Compared with the control macrophages, Dpep2-deficient macrophages exhibited much more enrichment of p-p65 as early as 15 min post CVB3 stimulation, and this superiority maintained to 4 h. Consistently, the upstream signaling molecules IKK and IκB were also more robustly activated in Dpep2-deficient macrophages (Figure 6), indicating that Dpep2 could efficiently suppress IKK-IκB-NF-κB signaling pathway and lead to the restrained inflammation in macrophages.

## Discussion

Viral myocarditis is a common heart disease characterized by cardiac inflammation and primarily mediated by excessive inflammatory responses. In physical condition, inflammatory responses are orchestrated and regulated precisely by both signals that evoke and maintain inflammation as well as signals that terminate this course. However, this delicate balance can be disturbed by virus infection, which can further lead to uncontrolled inflammation as well as consequent cellular and tissue damages. Macrophages are the earliest infiltrating and the main subset of cardiac inflammatory cells at the early stage of the CVB3-induced VMC (Fairweather and Rose, 2007). Except for clearing and presenting the damaged cardiomyocytes to elicit adaptive immune responses, macrophages could also modulate the cardiac immune microenvironment by producing cytokines, chemokines as well as growth factors (Lane et al., 1992; Tanaka et al., 2001; Gordon and Martinez, 2010; Liu et al., 2014), and play an essential role in the inflammation initiation, augmentation as well as maintenance of inflammation (Epelman et al., 2015). Therefore, many attempts to manipulate macrophage inflammation or polarization have been carried out including the identification of new macrophage immune regulators (Ter Horst et al., 2015; Brady et al., 2016).

Dpep2, highly expressed in the lungs, heart and testes (Habib et al., 2003), was originally identified as a membrane-bound dipeptidase that hydrolyzes LTD4 to LTE4. Recent studies showed that Dpep2 expression altered in the temporal fossa arachnoid cysts (Aarhus et al., 2010), implying that Dpep2 might be involved in the inflammatory responses. However, whether Dpep2 was involved in CVB3-induced myocarditis was still unknown. In this study, we found that cardiac Dpep2 expression was significantly increased in the CVB3-induced myocarditis, indicating that Dpep2 participated in the disease development. Moreover, cardiac Dpep2 was primarily enriched in the cardiac infiltrating macrophages but not the infected cardiomyocytes, indicating that the potential role of Dpep2 on macrophage inflammation during VMC. This was further confirmed by the more abundant pro-inflammatory cytokine production in CVB3-stimulated Dpep2-deficient BMDMs as well as more severe VMC in macrophage-specific Dpep2-deficient mice. Actually, previous studies had provided some clues to the association of Dpep2 with macrophages, in which they reported that Dpep2 may be one characteristic of activated macrophages (Oliveira et al., 2010).

As a dipeptidase, Dpep2 can hydrolyze LTD4 to yield LTE4. Both the substrate and product have been reported to be associated with inflammatory diseases (Habib et al., 2003; Shirasaki et al., 2015; Bankova et al., 2016), especially LTE4, which could promote the anti-inflammatory molecule cAMP production (Steinke et al., 2014). It suggested that LTE4 might involve in the macrophage inflammatory responses. However, we observed that exogenous LTE4 barely reduced the pro-inflammatory cytokine production by CVB3-stimulated Dpep2-deficient macrophages. Since transcription factor nuclear factor-κB (NF-κB) regulates a lot of immune and inflammatory genes (Oeckinghaus and Ghosh, 2009), and its pro-inflammatory function has been widely reported in macrophages (Lai et al., 2018; Liu et al., 2018), here we tried to explore the possible role of Dpep2 on macrophage NF-κB signaling pathway. We found that Dpep2 deficiency could significantly enhance the activation of p65, IκBα and IKKα in CVB3-stimulated macrophages, indicating that Dpep2 could efficiently inhibit macrophage NF-κB pathway. So far, we are still not clear about how this dipeptidase inhibited NF-κB signaling, while previous studies reported that other dipeptidases, such as dipeptidase carnosinase (CNDP1) and cellular non-specific dipeptidase (CNDP2) might be associated with oxidative stress as well as the subsequent inflammation (Yamakawa-Kobayashi et al., 2017), and dipeptidyl dipeptidase DPP9 could act as a novel endogenous inhibitor of NLRP1 inflammasome (Zhong et al., 2018). All these works indicated that some dipeptidases possess the properties to modulate the intracellular inflammatory signaling pathways, and this might provide us with some clues to explore the immune-modulation mechanism of Dpep2 on macrophage inflammation. Whether Dpep2 could modulate macrophage NF-κB signaling pathway via impacting oxidative stress or other process needs to be further studied.

Meanwhile, we noticed that the Dpep2 expression in the cardiac macrophages was peaked at day 3 post-infection (Figure 2A), of note, at the same time the cardiac viral load also achieved the maximum (Fairweather and Rose, 2007), this coincidence could be explained by the inflammation inhibition effect of macrophage Dpep2 facilitated the virus replication. As time went by, macrophages no longer represented the primary subset of cardiac infiltrating inflammatory cells, lymphocytes (T and B cells) and neutrophils instead constituted the majority of infiltrating cells, and of note obviously up-regulated Dpep2 could also be evidenced in the cardiac T cells, B cells and neutrophils (data not shown). By now herein we showed the Dpep2's inflammation inhibition effect in macrophages, while their impacts on T cell, B cell and neutrophil immune responses were still unclear, and Dpep2 might perform other immune impacts but not inflammation inhibition function in these cells. This might explain why the increased Dpep2 expression in the whole heart tissue did not efficiently repress the overproduction of pro-inflammatory cytokines in CVB3-induced myocarditis. This inspired us that abundant macrophage Dpep2 at the early stage of VMC might be important for controlling massive cardiac inflammation, and ensuring competent Dpep2 in macrophages might represent a novel therapeutic strategy against CVB3-induced myocarditis.

In summary, our results revealed a novel inflammation inhibition effect of macrophage Dpep2 in the context of CVB3-induced myocarditis, and showed that in addition to being a dipeptidase, macrophage Dpep2 could also modulate cardiac inflammatory responses and alleviate CVB3-induced myocarditis by acting as a regulator of NF-κB inflammatory signaling pathway. Our findings might shed light on the pathogenesis of CVB3-induced myocarditis, and may provide a potential treatment strategy for VMC based on manipulating macrophage Dpep2 expression.

## Data Availability

Publicly available datasets were analyzed in this study. This data can be found here: https://www.ebi.ac.uk/arrayexpress.

## Author Contributions

SX conceived the study. YY designed the experiments. XY performed the experiments, analyzed the results, and wrote the manuscript.

### Conflict of Interest Statement

The authors declare that the research was conducted in the absence of any commercial or financial relationships that could be construed as a potential conflict of interest.
